# Absolute quantification and characterization of oxylipins in lupus nephritis and systemic lupus erythematosus

**DOI:** 10.3389/fimmu.2022.964901

**Published:** 2022-10-06

**Authors:** Jingquan He, Chiyu Ma, Donge Tang, Shaoyun Zhong, Xiaofang Yuan, Fengping Zheng, Zhipeng Zeng, Yumei Chen, Dongzhou Liu, Xiaoping Hong, Weier Dai, Lianghong Yin, Yong Dai

**Affiliations:** ^1^ Department of Radiotherapy, Shenzhen Traditional Chinese Medicine Hospital, The Fourth Clinical Medical School of Guangzhou University of Chinese Medicine, Shenzhen, China; ^2^ Clinical Medical Research Center, Guangdong Provincial Engineering Research Center of Autoimmune Disease Precision Medicine, Shenzhen Engineering Research Center of Autoimmune Disease, The Second Clinical Medical College of Jinan University, The First Affiliated Hospital of Southern University of Science and Technology, Shenzhen People’s Hospital, Shenzhen, China; ^3^ Biotree Metabolomics Research Center, Biotree, Shanghai, China; ^4^ College of Natural Science, University of Texas at Austin, Austin, TX, United States; ^5^ Department of Nephrology, The First Affiliated Hospital of Jinan University, Guangzhou, China

**Keywords:** systemic lupus erythematosus, lupus nephritis, polyunsaturated fatty acids, oxylipin, SLEDAI

## Abstract

Systemic lupus erythematosus (SLE) is a chronic autoimmune disease with multi-organ inflammation and defect, which is linked to many molecule mediators. Oxylipins as a class of lipid mediator have not been broadly investigated in SLE. Here, we applied targeted mass spectrometry analysis to screen the alteration of oxylipins in serum of 98 SLE patients and 106 healthy controls. The correlation of oxylipins to lupus nephritis (LN) and SLE disease activity, and the biomarkers for SLE classification, were analyzed. Among 128 oxylipins analyzed, 92 were absolutely quantified and 26 were significantly changed. They were mainly generated from the metabolism of several polyunsaturated fatty acids, including arachidonic acid (AA), linoleic acid (LA), docosahexanoic acid (DHA), eicosapentanoic acid (EPA) and dihomo-γ-linolenic acid (DGLA). Several oxylipins, especially those produced from AA, showed different abundance between patients with and without lupus nephritis (LN). The DGLA metabolic activity and DGLA generated PGE1, were significantly associated with SLE disease activity. Random forest-based machine learning identified a 5-oxylipin combination as potential biomarker for SLE classification with high accuracy. Seven individual oxylipin biomarkers were also identified with good performance in distinguishing SLE patients from healthy controls (individual AUC > 0.7). Interestingly, the biomarkers for differentiating SLE patients from healthy controls are distinct from the oxylipins differentially expressed in LN patients *vs.* non-LN patients. This study provides possibilities for the understanding of SLE characteristics and the development of new tools for SLE classification.

## Introduction

Systemic lupus erythematosus (SLE) is an autoimmune disease characterized by uncontrolled autoantibody production, chronic systemic inflammation, and multi-organ damage (1). The destroy of end-organs and the subsequent death of SLE patients were largely due to the immunity alterations and sustained inflammatory effects (2, 3). Therefore, understanding the molecule signatures contributing to SLE pathogenesis, especially those involved in immunity and inflammation, will prompt the development of therapeutic and diagnostic tools.

Recent studies have provided new insights on the molecule mediators linked to SLE pathogenesis, as well as the molecule signatures for the development of new strategies for clinical diagnosis (4–6). The well-characterized autoantibodies, including antinuclear antibodies (ANA), are widely used for serology detection of SLE, classification of disease activity and monitoring of prognosis (7, 8). A series of pro-inflammatory or immunomodulatory cytokines, such as IL-1, IL-6, IL-10, TNF, IFN-I and BAFF, are highly upregulated in SLE, and related to the autoantibody deposition in different organs (9, 10). In addition, the mRNA expression profiles, even at single cell sequencing level, have provided new opportunities for the understanding SLE immunity features (11, 12). Furthermore, some small metabolites are also identified as immunity and inflammation modulatory mediators in SLE, such as sex steroids (13). However, these findings are still not enough to uncover the mysteries of SLE, thus limited the development of new tools for clinical usage.

Oxylipins are a new class of lipid mediators produced through either enzymatic or non-enzymatic oxidations of polyunsaturated fatty acids (PUFAs) (14). The well-known PUFAs are linoleic acid (LA), α-linolenic acid (ALA), arachidonic acid (AA), docosahexanoic acid (DHA), eicosapentanoic acid (EPA) and dihomo-γ-linolenic acid (DGLA). Oxylipins play very important roles in physiological and pathological conditions, especially in the regulation of inflammation and immune responses (15, 16). Dysregulation of oxylipin metabolism was observed in many diseases, including cancer, cardiovascular diseases, aging and rheumatoid arthritis (17–19). Many drugs targeting the oxylipin metabolic enzymes were developed and some clinical trials associated with oxylipin metabolism also showed promising prospects (19). In SLE, several of oxylipins had been identified to be significantly changed and critical for SLE pathophysiology, including epoxyeicosatrienoic acid (EET), Thromboxane A2 (TXA2), prostaglandin E2 (PGE2) and PGD2 (20–23), suggesting potential utility of oxylipins as candidate biomarkers for SLE diagnosis and clinical treatment. However, there’s still no detailed overview on global oxylipins expression profiles in SLE and whether they were good candidates for clinical usage.

In this study, a novel mass spectrometry-based method was used to absolutely quantify oxylipins and screen their global alterations in serum of SLE patients. Further analyses were conducted to evaluate the potential role of oxylipins in SLE diagnosis and SLE disease activity monitoring.

## Materials and methods

### Study cohort

This study was approved by the Ethical Committee of Shenzhen People’s Hospital in compliance with the Declaration of Helsinki (Approval No. LL-KY-2019365). All volunteers provided informed consent.

Our study included a total of 98 SLE patients who meet the European League Against Rheumatism/American College of Rheumatology (EULAR/ACR) 2019 classification criteria (24). All patients were recruited from the inpatient center of Shenzhen People’s Hospital from January 2019 to August 2019. The average age of SLE patients was 36.87 ± 1.21 years old (94.90% female). Among them, 33 were diagnosed with lupus nephritis by renal biopsy. In addition, 106 people with comparable age (34.50 ± 1.01 years old) and gender (84.11% female) and not diagnosed as an inflammatory autoimmune disease were included as healthy controls. To exclude the potential influence of glucocorticoid treatment on the expression of oxylipins, only the patients stopped taking glucocorticoids (Methylprednisolone or Prednisone) by themselves for at least three weeks before sample collection was included. Disease activity was assessed using the systemic lupus erythematosus disease activity index (SLEDAI) (25). The detailed clinical information of SLE patients was shown in **Table 1** and **Supplementary Table S1**. Serum samples from those participants were collected after overnight starvation, and all of the samples were collected prior to any treatment. After 1 h of stratification and centrifugation at 3000 rpm for 10 min at room temperature, the supernatants were divided to several aliquots and frozen in liquid nitrogen immediately for 15 min and put in -80°C for long-term storage. No sign of hemolysis occurred in all samples. One aliquot was used for oxylipin quantification.

**Table 1:** Clinical characteristics of the SLE patients at the time of recruitment.

	Healthy controls (n=106)	SLE (n=98)
**Basic information**		
Male (n, %)	17 (16.04%)	5 (5.10%)
Female (n, %)	89 (83.96%)	93 (94.90%)
Age (years)	34.50 ± 1.01	36.87 ± 1.21
BMI	21.37 ± 0.67	21.45 ± 0.40
TG (mM/L)	0.99 ± 0.10	1.41 ± 0.08
TC (mM/L)	4.59 ± 0.16	4.43 ± 0.13
ALT (U/L)	18.01 ± 1.03	17.19 ± 1.55
AST (U/L)	18.91 ± 0.59	19.10 ± 0.53
**Immunological domain**		
ANA (AU/mL)	–	2443.30 ± 202.82
Anti-dsDNA (IU/mL)	–	83.36 ± 10.72
C3 (g/L)	–	0.82 ± 0.03
C4 (g/L)	–	0.18 ± 0.01
**Clinical domain**		
SCr (μM/L)	–	85.32 ± 13.52
24h urine protein (g/24h)	–	0.85 ± 0.15
White blood cell (10^9/L)	–	6.04 ± 0.26
Platelet (10^9/L)	–	226.16 ± 7.97
Rash (%)	–	17 (17.35%)
Mucosal ulcer (%)	–	3 (3.06%)
Renal (Renal biopsy, %)	–	33 (33.67%)
Fever (%)	–	13 (13.27%)
Musculoskeletal (%)	–	26 (26.53%)
CNS (%)	–	5 (5.10%)
Serosal (%)	–	20 (20.41%)

BMI, body mass index; TG, triglyceride; TC, total cholesterol; ALT, alanine aminotransferase; AST, aspartate aminotransferase; ANA, anti-nuclear antibodies; Anti-dsDNA, anti-dsDNA antibodies; C3, complement protein C3; C4, complement protein C4; SCr, serum creatinine.

### Oxylipin extraction

Oxylipins were extracted by using SPE columns (Oasis^®^ PRiME HLB 1cc (30mg), Waters, USA) according to manufacturer’s instruction. Briefly, 200 μL serum samples from each individual were mixed with 400 μL methanol containing isotopically-labelled internal standards. Information about the internal standards, such as the retention time, collision energy, precursor and product ion and the concentrations added in each sample, was shown in **Supplementary Table S2**. Samples were then centrifuged at 12000 rpm for 15 min at 4°C. 400 μL supernatant were transferred out and mixed with 267 μL water. Samples were further purified with SPE cartridges. Finally, the samples were eluted with 1 mL methanol, and evaporated. Then they were reconstituted in 80 μL of 30% acetonitrile in water and subjected to further analysis. Quality control (QC) was prepared by mixing a small number of reconstitutions (10 μL each) and injected periodically during MS analysis.

### UHPLC-MS/MS analysis

An EXIONLC ultra high-performance liquid chromatography (UHPLC) (Sciex, Framingham, USA) coupled with a 6500 QTRAP+ triple quadrupole mass spectrometer (Sciex) was used for oxylipin analysis. An ACQUITY UHPLC BEH C18 column (Waters, Massachusetts, USA) was used to separate samples. Mobile phase A consisted of 0.01% formic acid in water, and mobile phase B consisted of 0.01% formic acid in acetonitrile. Column temperature was set at 50°C. Auto-sampler temperature was set at 4°C and the injection volume was 10 μL. Ion source parameters were as follows: curtain gas as 40 psi, IonSpray Voltage as -4500 V and Ion Source Gas as 30 psi. All the multiple reaction monitoring (MRM) data acquisition and processing were done by using Sciex Analyst Workstation Software (Sciex, v1.6.3) and Multiquant software (Sciex, v3.03). The standard solutions of all individual analytes were injected into the API source of the mass spectrometer, and the MRM parameters were optimized. For each Q1/Q3 (precursor ion/product ion) pair, the collision energy (CE) was also optimized. Then, the Q1/Q3 pairs with the highest sensitivity and selectivity were selected as ‘quantifier’ for quantitative monitoring (**Supplementary Table S3**). The absolute concentration of each oxylipin was calculated by comparison to the standard curves generated through mass spectrometry measure of series dilutions of all 128 oxylipin standards. Only the oxylipins with a signal to noise ratio greater than 10 (S/N>10) and the concentration located in the range of the standard curves were considered as absolutely quantified. The accuracy and precision of the current method was validated for linearity of standard curve, analyte recovery rate and relative standard deviation (RSD).

### Metabolic activity analysis

To quantify metabolic activity of AA, LA, ALA, DGLA, EPA and DHA, all the oxylipins were first classified to different metabolic pathways or lipid sets according to the unsaturated fatty acid it was produced from. For example, all the oxylipins produced from AA were classified as one lipid set; all the oxylipins produced from LA were classified as another lipid set. After that, the absolute intensity of each metabolite was z-score normalized. Then, the average z-scores of all the corresponding metabolites in each pathway, representing the metabolic activity, were calculated if at least three metabolites in the pathway identified.

### Random forest classifier and ROC curves

To identify oxylipins for distinguishing SLE patients and healthy controls, random forest classification method was used (Metaboanalyst 4.0) (26). The feature ranking method was random forests built-in. The performance of the model was evaluated by receiver operating characteristic curve (ROC). After confirmation of the top and most important features, all the samples were randomly divided to two parts. The first two thirds were used to construct a new random forest model by using the selected most important features directly, and the one third of samples were hold out for model validation and new sample prediction. In the new sample prediction procedure, the recall and precision values were calculated by using the following formula. Recall = TP/(TP+FN), and Precision = TP/(TP+FP). Here, TP represents true positive, FP represents false positive, and FN represents false negative.

### Statistics

Statistical analysis was performed by using SPSS (v25.0, International Business Machines Corporation, New York, USA), SIMCA (Umetrics, Sweden) and R software (Foundation for Statistical Computing, Vienna, Austria). For comparison between SLE and healthy controls, student’s t test was used, and the oxylipins with |Fold change|>1.2 and pvalue<0.05 were considered as significantly changed. For comparison between LN and non-LN patients, student’s t test or rank-sum test was used. Multiple hypothesis testing was applied by using Benjamini-Hochberg method (Qvalue). The metabolic activity of each lipid set was calculated by averaging the z-score of each member metabolite in the lipid set (27). Principal component analysis (PCA) was conducted to evaluate the difference in oxylipin expression profile between SLE patients and healthy controls. The difference of oxylipin metabolic profile between two samples was evaluated by Euclidean distance. Spearman’s correlation was applied to evaluate the relationships between oxylipins and clinical information.

## Results

### MS-based quantification of oxylipins

We developed a mass spectrometry-based method for targeted and absolute quantification of 128 oxylipins and related metabolites (see Materials and Methods). In our method, a total of nine isotopic-labeled internal standards were added into all samples with a specific concentration (**Supplementary Table S2**) prior to oxylipin extraction. For accurate quantification, the internal standards were used for the correction of oxylipin extraction efficiency among different samples, the normalization of the concentration of each analyst and the monitoring of chromatographic responses (28).

All the 128 oxylipins were detected in negative ion mode, with each precursor ion had several product ions. However, only one product ion with highest sensitivity and selectivity was selected for the MRM detection. All the standard curves used for the quantification of each oxylipin were perfectly fitted with least squares method with the squared regression coefficient all above 0.996 (data not shown). Besides, for all the analytes, the average recovery rate was 101.3% (range from 93.0%-110.4%), the average RSD was 6.7% (range from 2.8%-12.3%). These data suggest a high degree of consistency and reproducibility of our measurement.

**Figure 1:**
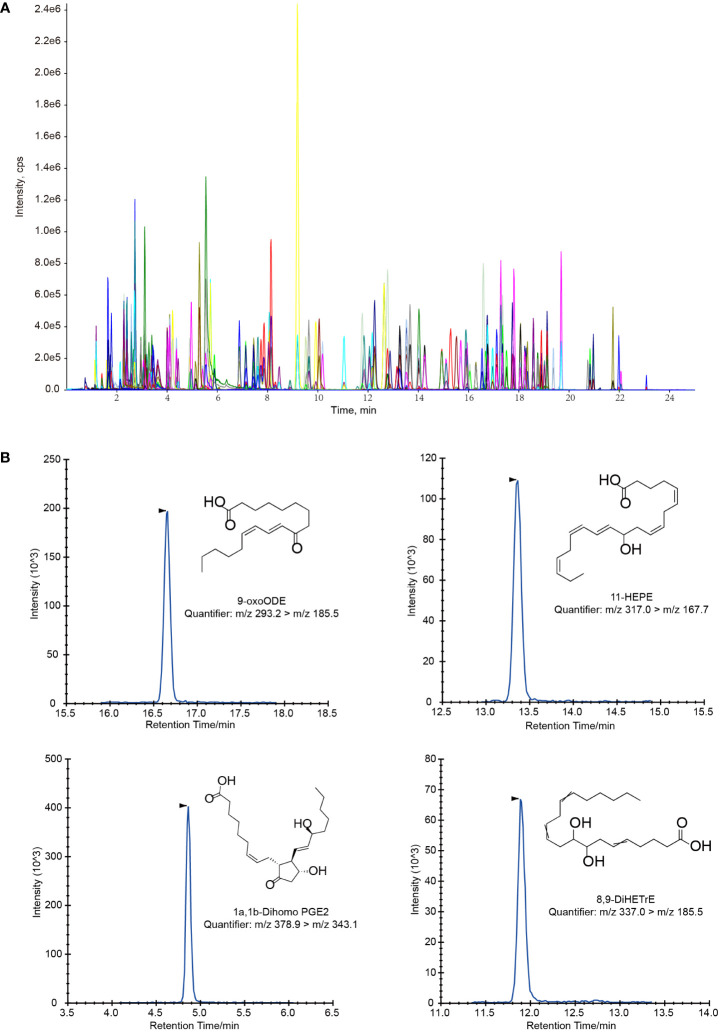
Extracted ion chromatograms (EIC) for oxylipin quantification. **(A)** LC-MS/MS chromatogram of a standard mixture of 128 oxylipins. **(B)** Several example of LC-MS/MS chromatograms of single oxylipin in magnified views.

A typical extracted ion chromatogram (EIC) for all the 128 oxylipins was shown in **Figure 1A**, and several EIC examples of single metabolite were also shown in **Figure 1B**. The MRM transition for all the 128 analysts was shown in **Supplementary Table S3**.

### Global alterations of oxylipins in SLE patients

**Figure 2:**
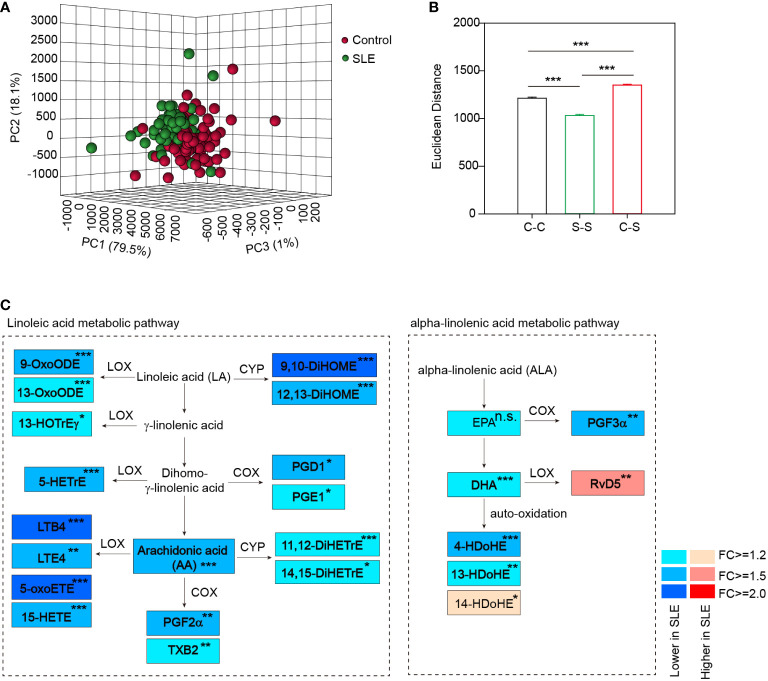
Distinct oxylipin expression profile in SLE patients. **(A)** PCA plot based on all 92 quantified oxylipins showed a separation of SLE patients and healthy controls. **(B)** Euclidean distance between SLE patients and healthy controls in the current cohort based on the global alterations of oxylipin abundance. C-C, distance within control group; S-S, distance within SLE group and C-S, distance between SLE and healthy controls. ***, p<0.001 by rank-sum test. **(C)** Diagram summarizing the concentration changes of oxylipins in linoleic acid metabolism and alpha-linolenic acid metabolism pathway. Significantly changed metabolites were indicated in rectangle with different colors. The fold changes of the altered metabolites in SLE patients were showed. *, p<0.05; **, p<0.01; ***, p<0.001. and n.s., non-significant. Linoleic acid (LA), γ-linolenic acid, Dihomo-γ-linolenic acid and α-linolenic acid (ALA) were not included in the 128 molecules analyzed. LOX, COX and CYP represent the enzymes that catalyze the formation of oxylipins.

Among the 128 analytes, 92 were absolutely quantified. They were mainly derived from polyunsaturated free fatty acids (PUFAs), including AA (n=48), LA (n=2), ALA (n=8), EPA (n=8), DHA (n=14), DGLA (n=9) and others (n=3) (**Supplementary Table S4**). Principal component analysis (PCA) based on all these oxylipins showed a separation between SLE patients and healthy controls (**Figure 2A**), suggesting the global difference in oxylipin metabolism. Besides, a larger oxylipin expression divergence between SLE and healthy controls was evident by using Euclidean distance compared with the difference within SLE patients or healthy controls (**Figure 2B**), further demonstrating the global oxylipin metabolism alteration in SLE patients. In the meantime, a smaller difference within SLE patients than healthy controls were also observed (**Figure 2B**). After that, we observed that 25 oxylipins were significantly changed (|Fold change|>1.2 and p<0.05 by student’s t test), and majority (92%, 23 out of 25) were decreased (**Supplementary Table S5**).

Oxylipins are mainly derived from alpha-linolenic acid and linoleic acid metabolic pathway in the action of three groups of enzymes, including lipoxygenase (LOX), cyclooxygenase (COX) and cytochrome P450 family proteins (CYP) (29), or by non-enzymatic auto-oxidation. We thus constructed a metabolic map detailing the alterations in the abundance of oxylipins and the corresponding catalytic enzymes in the two pathways (**Figure 2C**). We observed that several PUFAs were significantly decreased in SLE patients, including both the ω-6 PUFA (AA, >1.5-fold decrease) and the ω-3 PUFA (DHA, >1.2-fold decrease) (**Figure 2C**), which is similar to previous studies (30, 31). This was followed by the decrease of their downstream oxidized metabolites. For example, we observed a significant decrease of LTB4 (>2.0-fold decrease) in serum of SLE patients, which is consistent with a previous report (32). In addition, 5-oxoETE and 9,10-DiHOME were also ranked as the top significantly changed oxylipins with more than 2-fold decreases (**Figure 2C**).

### Oxylipins associated with lupus nephritis

Lupus nephritis (LN) is one of the most common and severe manifestations of SLE patients. It is the most important factor affecting the mortality of SLE patients. Previous reports have shown that oxylipins, such as EET and PGD2, play very important role in the progression of LN (20, 22). Thus, we analyzed the alterations of oxylipins in patients with LN (LN, 33 patients) compared with SLE patients without LN (non-LN, 65 patients) (**Table 1**).

**Figure 3:**
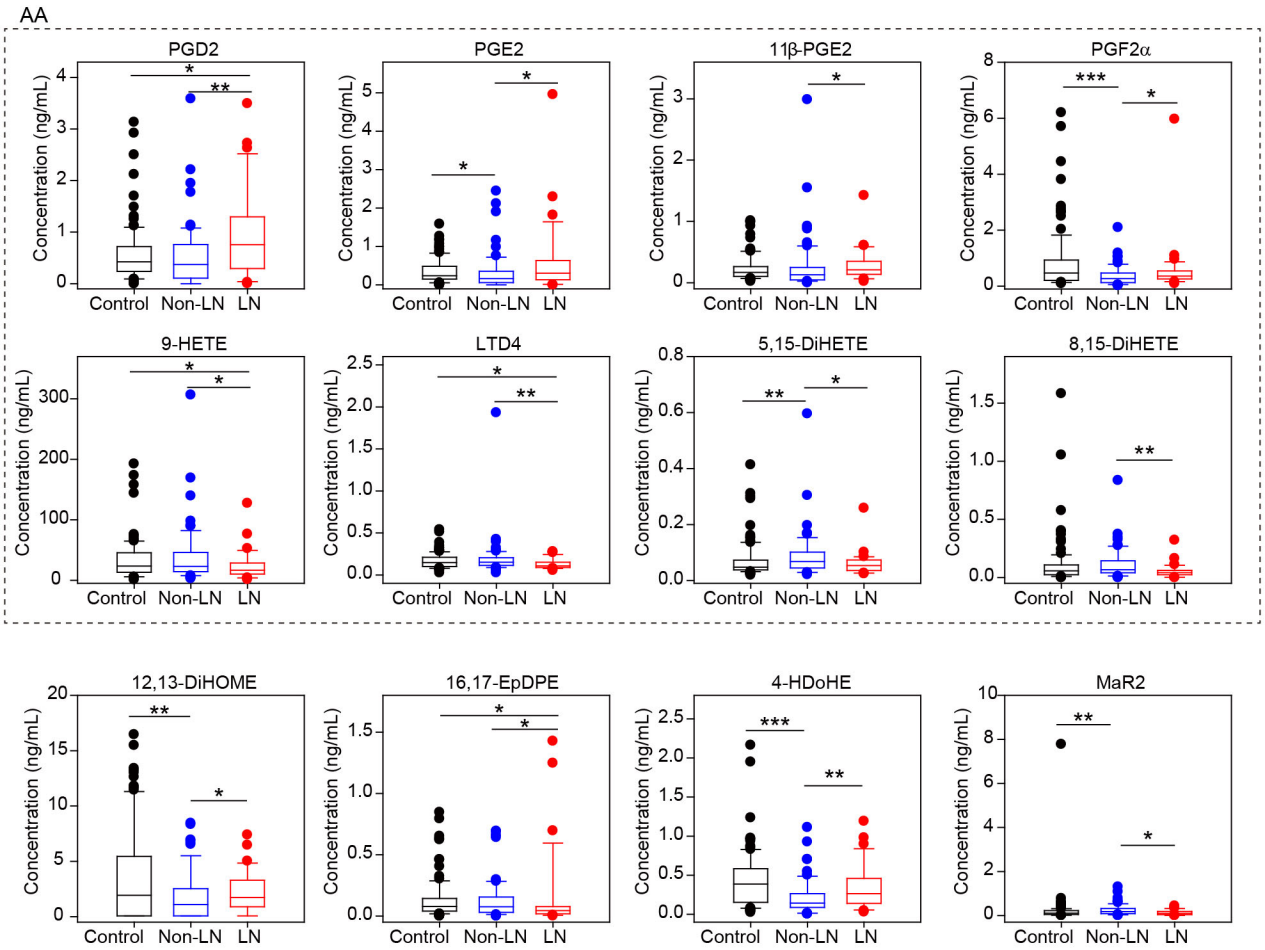
Different oxylipin metabolism between LN and non-LN patients. Box plots of 12 significantly different oxylipins between LN (n=33) and non-LN (n=65) patients. Dots represent outliers in each group. Control group is also included in this plot. The eight oxylipins in dashed box were all produced from AA. *, p-value<0.05; **, p-value<0.01 and ***, p-value<0.001 by student’s t test or rank-sum test.

After statistical analysis, we found that the abundance of 12 oxylipins in LN patients was significantly different from non-LN patients (P-value<0.05 by student’s t test or rank-sum test) (**Supplementary Table S6**), and eight of them were derived from AA (**Figure 3**). Among them, the concentration of PGD2, PGE2, 11β-PGE2 and PGF2α, were higher in LN patients, while the concentration of 9-HETE, LTD4, 5,15-DiHETE and 8,15-DiHETE were lower (**Figure 3**). Consistent to our data, PGD2 has been shown to be increased in the plasma of patients with SLE, and it amplified lupus-like disease and contributed to auto-antibody mediated kidney damage through CXCR4 (22). In addition, we also noticed that PGD2, PGE2, 11β-PGE2 and PGF2α were generated from the catalytic action of COX, but 9-HETE, LTD4, 5,15-DiHETE and 8,15-DiHETE were from LOX and CYP. These data suggested the upregulation of COX activity but downregulation of LOX and CYP activity for AA oxidization, which could be critical for the pathogenesis of LN.

### Oxylipins correlated to SLE disease activity

**Figure 4:**
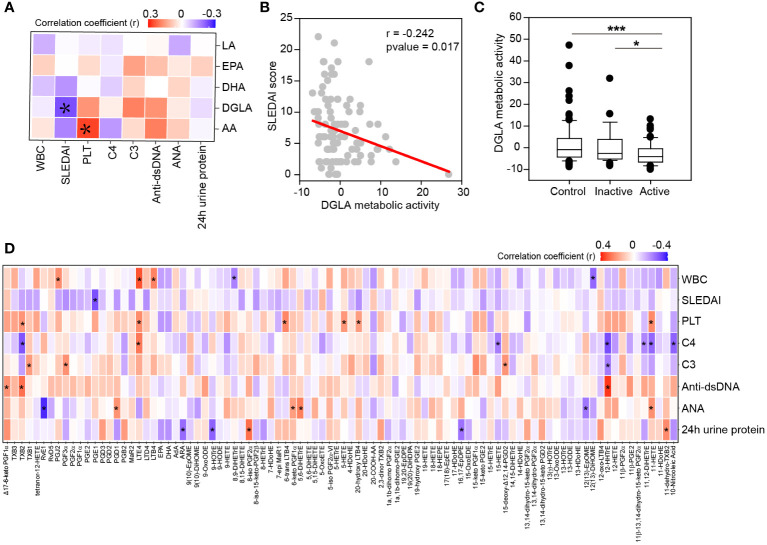
Clinical relationship of oxylipins with disease activity descriptors. **(A)** Heatmap of correlation matrix between PUFAs metabolic activity and disease activity descriptors. Significant correlations were labeled. Red, positive correlation; Blue, negative correlation. PLT, platelet count; WBC, leukocyte count. *, p-value<0.05. **(B)** Correlation of DGLA metabolic activity with SLEDAI score by linear regression. The correlation coefficient (r) and p-value were also showed. **(C)** DGLA metabolic activity among active patients, inactive patients and healthy controls. *, p-value<0.05 and ***, p-value<0.001 by rank-sum test. **(D)** Spearman’s correlation between individual oxylipin and disease activity descriptors. Red, positive correlation; Green, negative correlation. *, p-value<0.05.

Since SLE disease activity monitoring is critical for therapeutic decisions, it is important to understand the molecules associated with disease activity. We thus analyzed the relationship of oxylipins with disease activity descriptors. Oxylipins were first classified to different metabolic pathways according to the polyunsaturated fatty acids they originated from (**Supplementary Table S4**). Spearman’s correlations analysis was conducted, and the results showed that the metabolic activity of DGLA was negatively associated with SLEDAI (**Figure 4A**). The linear regression curve between DGLA metabolic activity and SLEDAI score was plotted in **Figure 4B**. Besides, a reduction of DGLA metabolic activity was also observed in active patients compared with healthy controls and inactive patients (**Figure 4C**). In the meantime, we also observed a positive correlation of AA metabolic activity to platelet counts (**Figure 4A**). These data suggest that AA and DGLA metabolism may contribute to the SLE disease development.

To uncover the exact molecules relating to SLE disease activity, the relationship between individual oxylipin and disease activity descriptors was determined by spearman’s correlation (**Figure 4D**). Many associations were found between oxylipins and complement proteins, auto-immune antibodies, 24 h urine proteins and blood cells. For example, leukotrienes (TXB2, LTE4, 6-trans LTB4 and 20-hydroxy LTB4) and HETEs (5-HETE and 11-HETE) were positively correlated to platelet counts. 12-HHTrE was positively correlated to anti-dsDNA antibody, but negatively correlated to complement protein C3 and C4. Besides, PGE1, one of the oxidative products of DGLA, was found to be significantly associated with SLEDAI score (r = -0.302, p-value = 0.002).

### Classification of SLE by oxylipins

**Figure 5:**
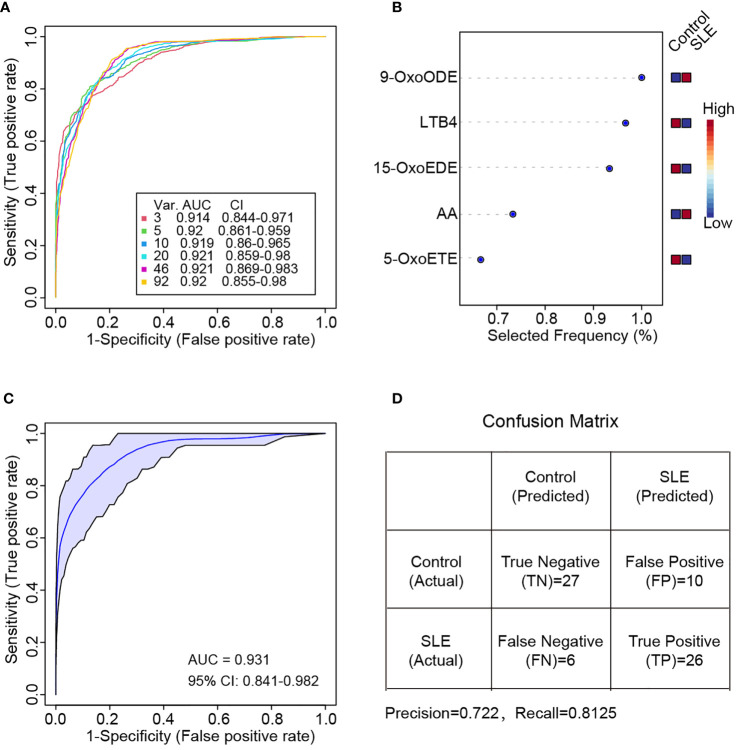
Random forest-based classification of SLE patients. **(A)** ROC curve of different oxylipin combinations for SLE classification. Six curves (3, 5, 10, 20, 46 and 92 features respectively) and the corresponding AUC and 95% confidence interval (CI) were shown. **(B)** Selected frequency of the top 5 most important features in random forest modeling. **(C)** ROC curve based on the five most important features in **(B)** Two thirds of samples were used for random forest modeling by directly using the five most important features in **(B, D)** Confusion matrix for the prediction of the one third hold out samples. The precision and recall value also calculated.

To identify the specific oxylipins that were able to distinguish SLE patients from healthy controls, random forest-based machine learning was applied and the top 3, 5, 10, 20, 46 and 92 features were selected to build classification models (26). ROC analysis was performed on different biomarker combinations. As it was showed in **Figure 5A**, by using three or five features, it can achieve pretty good performance. Using more features for SLE classification didn’t achieve better performance. The area under the curve (AUC) for three features and five features combination were 0.914 and 0.920 respectively (**Figure 5A**). These top molecules included 9-OxoODE, LTB4, 15-OxoEDE, AA and 5-OxoETE (**Figure 5B**).

Then, we divided these patients to two parts. The first two thirds were used to generate a new random forest model by using the top 5 oxylipins in **Figure 5B** directly, and the other one third samples were hold out for new sample prediction. The new model achieved even better performance of AUC = 0.931 (**Figure 5C**). For the new sample prediction, a confusion matrix was provided. The precision and recall were 0.722 and 0.8125 respectively (**Figure 5D**).

**Figure 6:**
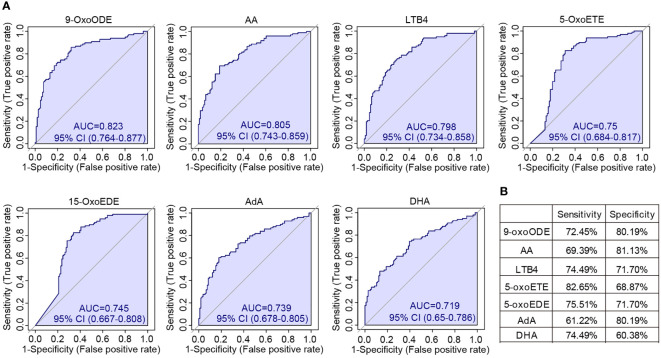
Univariate ROC analysis of individual oxylipins. **(A)** ROC curves showed all the oxylipins with AUC > 0.7 for the classification of SLE patients. The AUCs for different oxylipins were 0.823 (9-OxoODE), 0.805 (AA), 0.798 (LTB4), 0.75 (5-OxoETE), 0.745 (15-OxoEDE), 0.739 (AdA) and 0.719 (DHA). **(B)** The sensitivity and specificity of each seven individual oxylipin biomarkers for the detection of SLE.

We were also wondering that whether individual oxylipin was able to achieve high accuracy for SLE classification. To solve this, the univariate ROC analysis was conducted on each of the individual oxylipins. The results showed that seven molecules achieved high performance with AUC > 0.7. These included 9-OxoODE (AUC = 0.823), AA (AUC = 0.805), LTB4 (AUC = 0.798), 5-OxoETE (AUC = 0.750), 15-OxoEDE (AUC = 0.745), AdA (AUC = 0.739) and DHA (AUC = 0.719) (**Figure 6A**). The sensitivity and specificity of the seven molecules in distinguish SLE patients from healthy controls were shown in **Figure 6B**. Among them, 9-OxoODE, LTB4 and 5-OxoEDE had a sensitivity and specificity both greater than 70%.

## Discussion

In this study, we performed a novel mass spectrometry-based analysis and absolutely quantified 92 oxylipins in serum of SLE patients for the first time. We detected a global change of oxylipins in SLE patients (**Figure 2**). Similar alterations were also observed in previous reports, such as the decreased release of leukotriene B4 (LTB4) and AA in blood cells in SLE patients (32) (33), which can be used as an independent validation of our platform and results. Then we found that several oxylipins showed different abundance in LN patients compared to non-LN patients (**Figure 3**). Furthermore, several oxylipins, especially the metabolites of DGLA, were found to correlate with SLE disease activity (**Figure 4**). Finally, we also identified several oxylipins as novel biomarkers with good performance in differentiating SLE patients from healthy controls (**Figures 5**, **6**).

In this analysis, we only recruited the patients who were not taking steroids for at least 3 weeks. We have to mention that among the 98 SLE patients, 14 were diagnosed as SLE for the first time and had never taken any drugs. We compared the abundance of oxylipins between the patients who had a history of steroids treatment (n = 84) and the patients who had never taken steroids (n = 14). We found that only 9-HOTrE was significantly different [fold change (Steroids/Non-steroids) = 0.465, pvalue = 0.0395 by student’s t test]. This result demonstrated that oxylipin expression was not affected in the current patient cohort by steroids. However, since the sample size between the two groups was imbalance which will affect the statistical results, we didn’t show the data. More investigations on the impact of steroids on oxylipins need to be done in future. Besides, we also noticed that the level of blood triglycerides was higher in SLE patients than healthy controls (**Table 1**). This is a clinical feature of SLE patients as reported by previous research (34). Even though, to figure out whether triglycerides have impact on oxylipin level, general linear model was also constructed. Our results showed that most of the significantly changed oxylipins shown in **Figure 2C** were still significantly different between SLE and healthy controls, except TXB2, RvD5 and 14-HDoHE (data not shown), suggesting minimal effect of triglycerides on oxylipin expression.

It is well-known that oxidative stress is increased in patients with SLE, which contribute to the pathogenesis of SLE (35). Erasing of oxidative stress by potential antioxidant, such as N-acetylcysteine and conjugated linoleic acid, alleviated oxidative stress and induced disease remission (36, 37). To achieve this, the production of oxylipins by the oxidation of PUFAs would be one of the candidate pathways to target. In the quantification experiments, concentrations of AA, EPA and DHA were found to be significantly decreased in SLE patients (**Figure 2**), suggesting the decreased antioxidant capability. In addition, SLE is characterized as an inflammatory disease with the increase of many pro-inflammatory mediators and decrease of anti-inflammatory molecules (9). Besides, PUFAs, including EPA and DHA, can suppress the production of inflammatory mediators including IL-1, IL-2 and TNF-α, and dietary supplementation of those lipids induced SLE disease remission (38). The decreased abundance of those PUFAs in SLE patients suggested reduced defense ability to inflammation. Besides, some other anti-inflammatory molecules, such as 9-oxoODE, 13-oxoODE and 15-HETE were also significantly decreased in patients with SLE (**Figure 2**), further amplify inflammation in SLE patients. It is thus very important to quantify their contribution to SLE pathogenesis, or to verify whether they could be used in SLE clinical management. More experiments such as *in vitro* cell experiments, *in vivo* animal model experiments or even clinical trials could be conducted in future.

LN is a leading cause of morbidity and mortality in patients with SLE. There’s still lack of biomarkers for the differentiation of patients with and without LN, and there’s still very lack of the studies about the mechanism of LN pathogenesis. Many studies adopted large-scale proteomics or metabolomics approaches, which are typically very suitable for high abundant molecules. Oxylipins, a class of molecule with low abundance in human body but with very important functions, are rare detected and quantified. Our study found that several oxylipins, especially in AA metabolic pathways, were significantly different between LN and non-LN patients. Among them, PGD2 has been reported previously in LN pathogenesis (22). In addition, our data suggested the difference of COX activity to LOX and CYP activities, which could be candidate biomarker for LN pathogenesis (**Figure 3**).

SLE disease activity reflects the severity of disease at a specific time, which is critical for the decision of therapeutic approaches or even dietary management. Except the previously reported DHA and EPA (39), DGLA metabolic activity and DGLA produced oxylipins, including PGE1, were found to negatively correlate with SLEDAI score in this study (**Figure 4**). The role of DGLA was not fully investigated but had been shown to be protective for cardiovascular disease and inversely associated with the risk of depression (40, 41), suggesting a potential beneficial effect. The decrease of DGLA metabolic activity along with SLE disease activity highlighted the possible role of DGLA metabolism in SLE and even other autoimmune diseases.

According to the suggestions of European League Against Rheumatism (EULAR)/American College of Rheumatology (ACR), the 2019 newly released SLE classification criteria still included many criteria items (24). Though it has increased sensitivity and specificity, it is still a time-consuming classification criterion and not convenient to conduct. The distinct expression profile of oxylipins between SLE patients and heathy controls raised a possibility that some of them could be potential candidates for disease classification. Interestingly, this study identified several oxylipin biomarkers that are capable to distinguish SLE patients from heathy controls (**Figures 5**, **6**). A 5-oxylipin combination was selected in random forest model and seven individual oxylipins were also selected in univariate ROC analysis. Among them, LTB4 was previously identified as a biomarker for SLE diagnosis (42). Worth to note, these biomarkers are distinct from the oxylipins differentially expressed between patients with LN and non-LN. Our data provided new possibilities for the development of SLE classification tools.

In summary, we absolutely quantified 92 oxylipins and revealed their global changes in serum samples of SLE patients. We further analyzed the oxylipins associated to LN and disease activity. Furthermore, we also found several potential oxylipin biomarkers for SLE classification. Our data provided potential opportunities for development of new tools for clinical diagnosis and daily disease management.

## Data availability statement

The original contributions presented in the study are included in the article/**Supplementary Material**. Further inquiries can be directed to the corresponding author.

## Ethics statement

This study was approved by the Ethical Committee of Shenzhen People’s Hospital. The patients/participants provided their written informed consent to participate in this study.

## Author contributions

YD designed and supervised the study. ZZ, FZ, YC, and LY collected the clinical samples and clinical measurement information. XH and LY analyzed the clinical data. JH, SZ, and XY performed the mass spectrometry analyses. JH, CM, and WD conducted bioinformatics analyses. JH, DT, DL, and YD interpreted the data. JH wrote the manuscript. WD, DT, DL, and YD edited the manuscript. All authors contributed to the article and approved the submitted version.

## Funding

The project was supported by the Science and Technology Plan of Shenzhen (No. JCYJ20200109144218597 and JCYJ20180302145337935), the Key Research and Development Program of Guangdong Province (No.2019B020229001), the Basic and Applied Basic Research Fund of Guangdong Province (No.2021A1515110250), Shenzhen Key Medical Discipline Construction Fund (No. SZXK011) and Shenzhen Fund for Guangdong Provincial High-level Clinical Key Specialties (No. SZGSP001).

## Acknowledgments

We thank all the volunteers participated in this study. We thank Shanghai Biotree Biomedical Biotechnology co., LTD for the assistance in oxylipin data acquisition.

## Conflict of interest

SZ and XY are employed by the company Shanghai Biotree Biomedical Biotechnology Co., Ltd.

The remaining authors declare that the research was conducted in the absence of any commercial or financial relationships that could be construed as a potential conflict of interest.

## Publisher’s note

All claims expressed in this article are solely those of the authors and do not necessarily represent those of their affiliated organizations, or those of the publisher, the editors and the reviewers. Any product that may be evaluated in this article, or claim that may be made by its manufacturer, is not guaranteed or endorsed by the publisher.
